# Anomaly Detection with Feature Extraction Based on Machine Learning Using Hydraulic System IoT Sensor Data

**DOI:** 10.3390/s22072479

**Published:** 2022-03-23

**Authors:** Doyun Kim, Tae-Young Heo

**Affiliations:** Department of Information & Statistics, Chungbuk National University, Cheongju 28644, Korea; doyun2516@gmail.com

**Keywords:** anomaly detection, hydraulic components, IoT sensor, Boruta algorithm, true negative rate, true positive rate, condition monitoring

## Abstract

Hydraulic systems are advanced in function and level as they are used in various industrial fields. Furthermore, condition monitoring using internet of things (IoT) sensors is applied for system maintenance and management. In this study, meaningful features were identified through extraction and selection of various features, and classification evaluation metrics were presented through machine learning and deep learning to expand the diagnosis of abnormalities and defects in each component of the hydraulic system. Data collected from IoT sensor data in the time domain were divided into clusters in predefined sections. The shape and density characteristics were extracted by cluster. Among 2335 newly extracted features, related features were selected using correlation coefficients and the Boruta algorithm for each hydraulic component and used for model learning. Linear discriminant analysis (LDA), logistic regression, support vector classifier (SVC), decision tree, random forest, XGBoost, LightGBM, and multi-layer perceptron were used to calculate the true positive rate (TPR) and true negative rate (TNR) for each hydraulic component to detect normal and abnormal conditions. Valve condition, internal pump leakage, and hydraulic accumulator data showed TPR performance of 0.94 or more and a TNR performance of 0.84 or more. This study’s findings can help to determine the stable and unstable states of each component of the hydraulic system and form the basis for engineers’ judgment.

## 1. Introduction

Hydraulic systems are applied in various industries regardless of scale utilizing the properties of liquid that can be delivered to all parts without the loss of applied pressure. As hydraulic systems are applied in various fields, problems that may arise can manifest in different ways in industrial applications. Fluids within hydraulic systems have generally low flammability, but if they leak in the form of a spray, they can become highly flammable and cause severe problems [1].

With an aircraft that is controlled by a hydraulic system, a problem is contained by controlling the contamination of the fluid inside the system [2]. Inefficiency may arise because of a lack of control of the performance of the hydraulic system, which may waste energy [3]. Therefore, it is necessary to manage the performance, problems, and risk factors of a hydraulic system.

Research and development are in progress to monitor the status of hydraulic systems continuously and to detect and control possible issues in advance to minimize the occurrence of delays and financial problems caused by investigating how to solve hydraulic system problems and providing solutions.

The hydraulic system is largely composed of an accumulator, cooler, pump, and valve, and the characteristics of each are as follows. The hydraulic accumulator of the hydraulic system operates through fluid and gas, and since the fluid is relatively incompressible compared to gas, it is ideal for power transmission. On the other hand, gas has the characteristic of compressing a small volume at high pressure compared to a fluid; thus, energy can be stored in the gas, and thereafter transferred to the fluid as the high-pressure stage is released.

The hydraulic cooler generates heat while converting and transferring energy in the hydraulic system. As the fluid’s temperature increases, internal wear of the system occurs, and the worn particles become unnecessary elements in the fluid and may damage other components. The hydraulic valve can control the fluid’s flow and its pressure by opening and closing. The hydraulic pump is a component that can transfer energy to the fluid and is the core of the hydraulic system.

If at least one of the hydraulic system’s components does not work properly, a problem may occur in the entire system. For example, if the generated heat is not cooled, heat is applied to the fluid, which can cause further damage due to aging and loss of lubricity. In addition, leakage inside the hydraulic system can degrade the performance and efficiency of the system, worsening the problem over time. Various measures and control methods have been proposed to reduce vibration or noise generated in the hydraulic system as a signal of abnormality or the occurrence of a problem [4]. Among them, condition monitoring can reduce human inspection requirements, detect errors before they escalate, reduce maintenance costs, and improve safety and reliability [5]. In addition, it can maximize productivity by minimizing system downtime [6].

As the structure of hydraulic system-related equipment becomes increasingly complex, functions are strengthened and the level of automation is increased; hence, subjective error diagnosis based on visual observation and disassembly reaches its limit. Error diagnosis technology has been continuously developed in line with the level of technological growth. Research on fault diagnosis using machine and deep learning is being actively conducted after diagnosing hydraulic system faults based on signal processing technology and mathematical models [7]. Especially in condition monitoring, high-dimensional datasets have overlapping information to characterize properties of faults. Recently, to construct high-dimensional datasets, an intrinsic mode function (IMF) has been extracted from a raw signal using empirical mode decomposition (EMD) among signal decomposition methods, and numerical features are estimated for each function. However, dimensional reduction is required to prevent low diagnostic performance and overfitting of machine learning models due to high-dimensional datasets [8]. For example, collect real-time high-dimensional measurements from multiple sensors to diagnose faults in internal combustion engines. In constructing a diagnostic model, dimension reduction is required by feature extraction of a high-dimensional dataset in order to separate sensitive information that can positively affect diagnostic results and computational efficiency [9].

Similar to a previous study, this work proposes a supervised learning-based linear discriminant analysis suitable for detecting various types of errors in a wide time range [10]. Robustness is added to the condition monitoring system by implementing the common errors of the raw signals (constant offset, drift, noise, and signal peak) of the sensors used in hydraulic systems [11]. In addition, if the model optimized through random forest is used, even with limited sensor data, it can be sufficient to classify the hydraulic system’s state and can reduce storage capacity and training time [12]. 

Statistical-based features were extracted from sensor data, which varied depending on whether the vibration data generated in the hydraulic system were in stable or unstable states, to identify differences based on these states [13]. When the classification algorithm was performed by extracting and using new features from vibration data with fast Fourier transform, classification accuracy close to 1 was achieved [14].

We reduced the dimension of observations through principal component analysis (PCA), diagnosed hydraulic valve failures using XGBoost, realized higher accuracy than random forest [15], and built a sensor sensitivity prediction model based on a convolution neural network (CNN), so that the prediction quality matched that of the previously built model [16]. Data related to anomaly and problem detection often have imbalanced target variables; therefore, some studies classify them after applying oversampling, undersampling, and ensemble methods [17].

To detect internal leakage faults that affect the hydraulic system’s dynamic performance and reduce energy efficiency, periodic data were analyzed through trained support vector classifier (SVC) to detect faults early [18]. In addition, a more efficient electricity production process was proposed by integrating the production process control system and IT system by designing a method to detect faults in the water pump early based on data measured by the control device through internet of things (IoT)-enabled predictive maintenance [19].

In previous studies, duplicate data were removed and new information was extracted by mapping data to a new dimension. However, in this study, feature extraction was performed based on the shape and distribution density characteristic of the raw signal to create a new combination of features and by selecting the upper features related to the components of the hydraulic system, which contributed to the improvement of classification performance and diagnosed the abnormalities and faults of each component of the hydraulic system further.

In addition, the performance evaluation of the anomaly detection model was performed by measuring accuracy, precision, true positive rate (TPR), and true negative rate (TNR) from the confusion matrix of classification. In particular, the stability of the hydraulic system was classified through TPR to prevent incorrect system suspension due to misclassification, and accidents were prevented in advance by determining defects in the hydraulic system early through TNR. It can also be proposed as a basis for judgment of engineers and used as a clear measure of judgment by decision-makers.

The structure of this paper is as follows. In the Data Section, sensor data for each hydraulic system component are introduced. Data preparation through data preprocessing, feature extraction, feature selection, and introducing methodologies and model evaluation metrics for anomaly and defect classification through model learning is described in the Study Design Section. The Results Section reveals the classification of abnormalities’ results, defects, and the selection of excellent models through the model evaluation index, and the Discussion Section identifies strengths and weaknesses through comparison with previous studies and suggests future research tasks. Finally, the study is summarized in the Conclusion.

## 2. Hydraulic System Cyclical Sensor Data

To minimize any additional damage that can occur due to a possible defect in the hydraulic system, we determine whether an anomaly has occurred based on vibration data. Vibration data in a fluidized mechanical system is greatly influenced by lubrication temperature and operating speed, so attention should be paid to test temperature and working speed [20]. Since the vibrations that occur when an abnormality or problem occurs differ from what is normal, abnormality detection is conducted based on this information. Vibration can occur when a series of mechanisms do not operate within the proper range of motion.

The data used in this study can be obtained from the UCI Machine Learning Repository [10]. Data were obtained experimentally using hydraulic test equipment.

**Figure 1:**
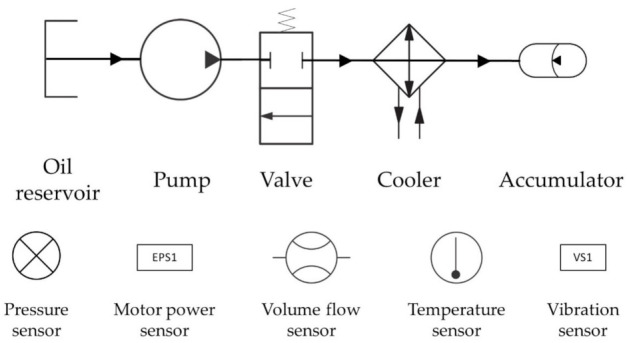
Concise hydraulic system circuit and installed sensors.

A hydraulic system circuit represents a set consisting of several hydraulic components. It controls the flow, position, and pressure of fluid based on circuit components such as hydraulic pumps, pipes, and hydraulic motors. Figure 1 describes a promised representation of a complex hydraulic system circuit for a more convenient view. It is easy to comprehend the hydraulic system circuit of the hydraulic test equipment that generates data using the promised representation in Figure 1. The hydraulic test equipment that generates the data proposed in this paper consisted of a primary circuit connected via an oil reservoir and a secondary cooling filtration circuit. In a working circuit with a main pump (electrical motor power of 3.3 kW), different load levels are cyclically repeated using a proportional pressure relief valve (V11) [15]. Experiments with predefined load levels are possible. The sensor data were collected from a PLC (Beckhoff CX5020) which transmitted the data to a PC via EtherCAT [16,21].

As the system repeated a cycle, it quantitatively changed the status of the four hydraulic components (accumulator, cooler, pump, and valve) presented in Table 1. Data was repeated 2205 times with a cycle of 60 s to change the status of the hydraulic system for each cycle. Furthermore, a binary value about stable flag indicated the presence or absence of an abnormal state according to the hydraulic system’s state corresponding to each cycle.

The pressure (PS1 to PS6, 100 Hz), motor power (EPS1, 100 Hz), volume flows (FS1 to FS2, 10 Hz), temperature (TS1 to TS4, 1 Hz), and vibration (VS1, 1 Hz) presented in Table 2 were obtained from the actual sensor. Cooling efficiency (CE, 1 Hz), cooling power (CP, 1 Hz), and system efficiency (SE, 1 Hz) were values measured by a virtual sensor. Therefore, 17 sensors’ data were measured in total.

## 3. Study Design

In this study, various analyses were conducted to detect problems and abnormalities in the hydraulic system. The data were pre-processed for correct data shape and feature extraction; the train/test set was separated to verify whether a correct model that was not overfitted to the training data was learned after model training. Appropriate information was extracted from the shape and data distribution of the sensor data, and the extracted features were selected using an appropriate algorithm and used as predictive variables for model training. The feature most related to the components of the hydraulic system was selected and used as a feature when learning the model.

**Figure 2:**
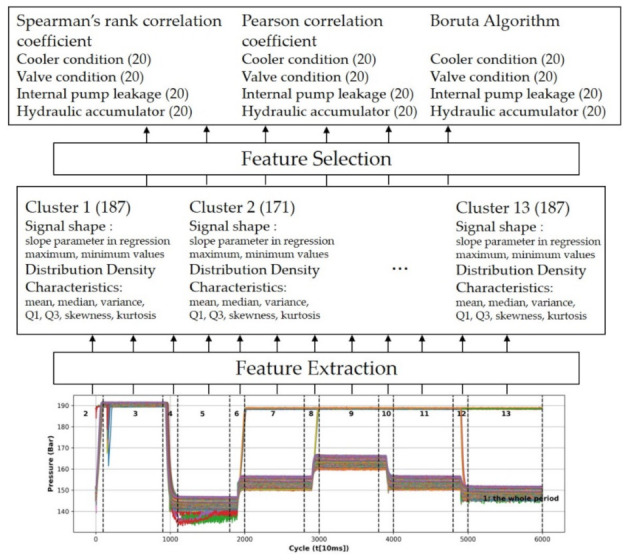
Data pre-processing, feature extraction, and feature selection for machine learning.

Figure 2 shows the process of extracting 187 or 171 new features per cluster from the data by feature extraction and selecting 20 features that explained the state of hydraulic components well by feature selection to select variables to use for the machine learning model.

### 3.1. Data Pre-Processing

**Figure 3:**
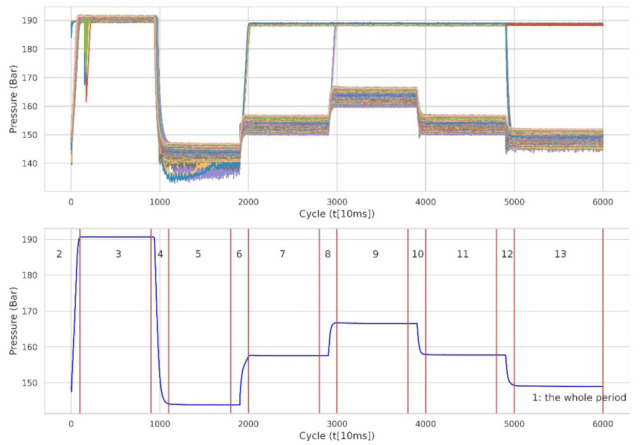
Pre-defined Clusters 1 to 13 for feature extraction.

The sensor data were measured 2205 times with a cycle of 60 s, and the number of vibrations measured for each repetition differed; hence, the data needed to be pre-processed. Therefore, to extract and select features that could be appropriately used for model learning through Figure 3, we divided the sensor data based on 13 clusters defined in advance based on PS1, which best describes a certain cycle. Cluster 1 corresponded to the entire cycle of FS1, and Clusters 2 to 13 were allocated in the divided order.

### 3.2. Feature Extraction

Feature extraction is a method to generate more suitable and useful features for model training through new combinations based on all features. It is important to use the full information we have, but if the appropriate information for model training exists locally, it can be used for model training, to result in a better model.

We extracted the slope of the linear fit, and the positions of maximum and minimum value for each cluster from the signal shape. Furthermore, the mean, median, Q1, Q3, variance, skewness, and kurtosis were extracted for each cluster from the distribution density characteristic. Additionally, since skewness and kurtosis were not calculated in Clusters 2, 4, 6, 8, 10, and 12 in which there was no gradient in the 1 Hz feature, in total 96 features were excluded, and 2335 new combinations of features were extracted.

#### 3.2.1. Signal Shape

Based on the cluster defined previously in the data-preprocessing step, the slope parameter of the linear regression and the positions of the maximum and minimum values were used as features for each cluster. The slope parameter of the linear regression with the locations of the maximum and minimum values is one of the methods to extract only the necessary information from our entire information and is particularly useful when the relevant information is included only within a specific period. Especially with linear regression, it is computationally very reasonable and provides a first-order approximation. If we used an even higher-order approximation, we could have information close to the original information, but unnecessary information such as noise may have been included, so we used the gradient as a feature [22].

#### 3.2.2. Distribution Density Characteristics

In past studies, time-domain features used statistics such as mean and variance to identify the difference between a general vibration signal and other vibration signals. Furthermore, skewness and kurtosis can be extracted through the probability density function of the vibration signal. Therefore, the sensor data of the hydraulic system are also composed of a time domain, and since it is data that collects vibration signals and repeats a certain period, distribution density characteristics (mean, median, variance, Q1, Q3, skewness, kurtosis) based on pre-classified clusters are extracted [13].

### 3.3. Feature Selection

Feature selection is a series of processes to improve model performance by reducing model computation cost and by reducing the number of features in developing a predictive model. The model is composed of the most relevant features among those recombined by feature extraction. Its advantage is that the number of dimensions can be managed and the model’s performance degradation can be prevented. The features most related to the state of the hydraulic system components described above were selected and used for model learning.

#### 3.3.1. Spearman’s Rank Correlation Coefficient

Since the Spearman rank correlation coefficient describes the relationship between two variables through a monotonic function between them, it evaluates a monotonic relationship regardless of its linearity. It can be expressed the same as the Pearson correlation coefficient of the rank values of two variables. The range of coefficients can have a value between −1 and 1, and it has a value close to 1 when observations between two variables have similar ranks, and has a value close to −1 when the ranks differ. An absolute value is applied to the calculated correlation coefficient to select a high correlation coefficient regardless of positive or negative correlation.

Table 3 presents the Spearman rank correlation coefficients for the type of feature extraction for each sensor for each hydraulic component and the cluster location, and selects the top 20 in absolute value order regardless of negative or positive values. For example, in the case of “FS1_maxloc_4”, which has the highest correlation coefficient of valve condition, the absolute value of the Spearman rank correlation coefficient with the valve condition in the 5th cluster of the FS1 sensor is 0.946.

#### 3.3.2. Pearson Correlation Coefficient

The Pearson correlation coefficient measures linear correlation between two variables, unlike the Spearman correlation coefficient. It can be expressed as the ratio between the covariance and the product of two variables. The range of the coefficient can have a value between −1 and 1, and the sign of the coefficient is determined according to the slope of the straight line. An absolute value is applied to the calculated correlation coefficient to select a high correlation coefficient regardless of positive or negative correlation.

Table 4 shows the Pearson correlation coefficient calculations for the type of feature extraction for each sensor for each hydraulic component, and the cluster location, and selects the top 20 in absolute value order regardless of negative or positive values. For example, in the case of “CE_Q1_13”, which has the highest correlation coefficient in the cooler condition, the absolute value of the Pearson correlation coefficient with the cooler condition in the 13th cluster of the CE sensor is 0.993.

#### 3.3.3. Boruta Algorithm

The Boruta algorithm is a random forest-based variable selection method. Existing variables that do not affect the model creation are shadow variables created by restoring and extracting existing variables. They are judged to be of insufficient value and are removed. The algorithm can be used without hyper-parameter tuning, and variable importance can be converted into numerical values.

Duplicate features are created for all features, and shadow features are mixed well and combined with existing features to eliminate correlation with response variables. Based on this, a random forest is executed to calculate the z-score. Among the shadow features, the feature with the highest z-score is marked as an important variable with +1 hit. By repeating the above process as many times as possible, random forest analysis is performed, and important and insignificant variables can be tagged.

**Figure 4:**
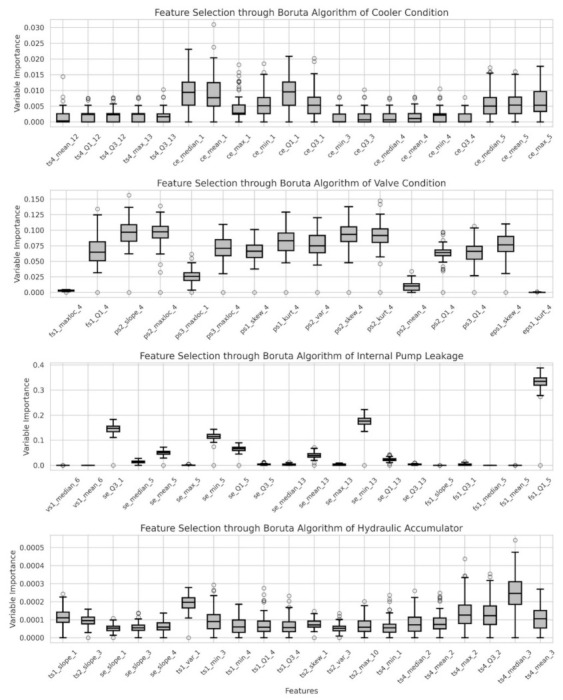
Feature selection through the Boruta algorithm.

Figure 4 shows the features selected by calculating the importance of features extracted for each hydraulic element using the Boruta algorithm. Twenty variables were selected for each component, and 16 variables were selected during feature selection of valve condition among hydraulic components through the Boruta algorithm.

### 3.4. Model Training

After completing the variable selection, a model for anomaly and defect classification was trained based on 20 features related to hydraulic components. The valve condition with feature selection with the Boruta algorithm trained a model based on 15 selected features. The response variable was a stable flag indicating the state of the hydraulic system for a total of 2205 repeated cycles. Linear discriminant analysis (LDA), logistic regression, support vector classifier (SVC), decision tree, random forest, XGBoost, LightGBM, and Multilayer perceptron were used as the methodologies to learn the anomaly and defect classification model. Furthermore, the optimal hyper-parameter was obtained using grid search as cross validation, and the optimization model was determined by preventing overfitting. After determining the model, its performance was evaluated by measuring accuracy, precision, recall, and the true negative rate.

#### 3.4.1. Linear Discriminant Analysis

LDA means that the discriminant function that categorizes an observation’s class is a linear function. In the existing dimension of the dataset, data are separated by projecting it to a smaller dimension that maximizes between-class variance and minimizes within-class variance.

Using each group mean $\mu_{c}$ and the total mean μ of our feature matrix, we find the between-group variance $\sum_{B}\;$ and within-group variance $\sum_{W}\;$, calculate the eigenvalues and eigenvectors of the transformation matrix $W=\sum_{W}^{-1}\sum_{B}\;$, and organize them according to the largest eigenvalues. The k th eigenvector from the first eigenvector means a new space $V_{k}$ of a smaller dimension. Finally, we project $X_{ij}$ into the new space $V_{k}$.

#### 3.4.2. Logistic Regression

In general, linear regression analysis is suitable for prediction but not for classification, because the response variable of linear regression analysis has no limit on the range of values. Therefore, the z value of the linear regression analysis $z=w^{T}x+b$ is substituted into the sigmoid function $y=\frac{1}{1+e^{-z}}=\frac{1}{1+e^{-\left(w^{T}x+b\right)}}$ to have a value between 0 and 1. The right side can be summarized by $w^{T}x+b$ and expressed in a linear state as shown in $log\left(\frac{y}{1-y}\right)=w^{T}x+b$. The left side was made into a logit that took a log to the odds ratio. Logistic regression analysis can be completed by replacing y in logit with π(x). The π(x) is P(y=1|X=x). The resulting value of logistic regression analysis is the classification probability, and the class is determined based on the classification threshold.

#### 3.4.3. Support Vector Classifier

The support vector classifier (SVC) determines the class based on the support vector. The SVC creates a straight line that separates each class. The SVC classifies the various divisible straight lines using a centerline and a support vector.

To draw a centerline, when the vector w is perpendicular to the centerline and the observation x is the dot product, the point at which the dot product value c becomes the centerline is $w^{T}x=c$. The data space can be divided into cases where the dot product is greater than c and less than c.

By transposing c to the left side and replacing −c with b, the centerline can be expressed as in Equation $w^{T}x+b=0$, which becomes a hyperplane that separates data, and Equation $G(x)=sign[w^{T}x+b]$ becomes an expression representing each region.

In the SVC, the margin, which is the width between support vectors, is maximized. The margin is maximized because the generalization error tends to be low. If the margin is small, the model may overfit.

$$max_{w,b}M\;subject\;to\;\{\begin{array}{c} y_{i}(w^{T}x_{i}+b)\geq M,\;\;\;\;i=1,\cdots ,n\\ |\left|w\right||=1 \end{array}$$

A soft margin through slack variables relaxes the existing SVC’s criteria to allow some misclassified observations. The kernel changes the feature space and applies it to the SVC. When the curved space is straightened after applying the SVC to it, the observations are classified well and the data are classified as a nonlinear decision boundary through the kernel. Kernel types include the polynomial, radial bias function, and sigmoid.

#### 3.4.4. Decision Tree

The decision tree can predict the class or value of the response variable by learning the decision rule from the training dataset. It starts at the root node to predict the class label using the decision tree. The internal node splits according to the properties of the root node. The leaf node is the node located at the bottom of the internal node. It contains the result of predicting the value of the class label or response variable and does not split any more. It has high enough interpretability to be interpreted without statistical knowledge, and there is no assumption about the type of data. It is not affected by multicollinearity and outliers.

#### 3.4.5. Random Forest

Random forest is an ensemble technique composed of multiple decision trees. Individual trees in the random forest predict a class, and the model predicts the specific class chosen by most classifiers. After randomly extracting n observations from observations so that overlap is possible, the process of creating a decision tree is repeated by selecting p features so that overlap is not possible. The final model prediction is made by voting or averaging the results from each decision tree.

Although it is relatively slow due to the characteristics of the random forest, individual decision trees differ, and since not all features are considered, the performance degradation of the model can be prevented in high-dimensional data.

#### 3.4.6. XGBoost

Boosting reduces the weight of correctly classified observations while learning, and assigns weights to difficult-to-classify observations to focus ultimately on the difficult-to-classify observations. The learner created in the previous step is used to change the weights of the dataset to be used in the next step.

Gradient boosting is a method to boost the use of boosting gradients, and it learns in the direction of reducing the residual obtained by comparing the predicted result obtained by including the feature into the learning model and the actual result. If the residual is large, a new model is added to improve the performance continuously by adding a new model that can reduce the difference between the existing predicted result and the actual result. XGBoost is a type of ensemble boosting technique, and has the advantage of fast model learning by parallel processing gradient boosting.

#### 3.4.7. LightGBM

The existing tree-based algorithm uses a level-wise method that splits while maintaining a balanced tree as much as possible. Although the depth of the tree can be minimized, it has a characteristic that requires additional time to balance. LightGBM has a leaf-wise tree partitioning method that extends vertically. Without considering the balance of the tree, it continuously divides the leaf node with the maximum loss to create a deep and asymmetric tree. LightGBM has maximum data loss, so it can minimize the prediction error loss compared to the level-wise tree split method and it takes relatively less learning time and uses less memory compared to XGBoost.

#### 3.4.8. Multilayer Perceptron

A multilayer perceptron consists of three or more node layers of input, hidden, and output layers. Except for the input layer, each node uses a nonlinear activation function. To train the MLP, the weights are changed using backpropagation, which reduces the error by reversing the error between the actual and predicted values. Multilayer and nonlinear functions can distinguish between linearly inseparable data. When all nodes are connected, there is a disadvantage that can lead to inefficiency by including too many parameters.

#### 3.4.9. Cross Validation

The ratio of the steady state to the unstable state of the target variable of the data analyzed is (7449, 756), and the ratio of the unstable state is about 10% compared to that of the steady state. To prevent errors, the cross validation used in our study is stratified k-fold to prevent a specific target variable from being concentrated on a specific fold.

Furthermore, the grid search method is used to select suitable hyper-parameters of the model with the best performance by sequentially inputting predetermined values to find the optimized model hyper-parameter.

### 3.5. Classification Evaluation Metrics

The evaluation indicators used for model evaluation are accuracy, precision, recall, and true negative rate. Precision is an evaluation index to measure positive prediction performance accurately as the ratio of actual positive observations among observations with positive prediction results. The recall is the ratio of positively predicted actual positive observations that is also called sensitivity or true positive rate (TPR). It is useful to identify issues that may be sensitive when a negative is actually positive. 

True negative rate (TNR) is a ratio that is correctly predicted to be negative among observations that are actually negative. Like the TPR, it is useful to identify problems that may be sensitive when a negative is actually positive. Since our study aims to gauge whether the hydraulic system is stable to prevent further damage, it is judged that the hydraulic system is in a stable state but unstable through recall (TPR) rather than accuracy, so we reduce the financial damage and reduce the actual unstable state through TNR. The goal is to reduce a state that is judged stable but could increase the damage.

$$\mathrm{Accuracy}\;=\frac{\mathrm{TP}\;+\;\mathrm{TN}}{\mathrm{TP}+\mathrm{TN}+\mathrm{FP}+\mathrm{FN}}\;\mathrm{Precision}\;=\frac{\mathrm{TP}}{\mathrm{TP}+\mathrm{FP}}\;\mathrm{Recall}\;=\;\mathrm{True}\;\mathrm{Positive}\;\mathrm{Rate}\;=\frac{\mathrm{TP}}{\mathrm{TP}+\mathrm{FN}}\;\mathrm{True}\;\mathrm{Negative}\;\mathrm{Rate}\;=\frac{\mathrm{TN}}{\mathrm{TN}+\mathrm{FP}}$$

## 4. Results

We selected the top 20 features that are highly related to each hydraulic system component and trained the model for the experiment. Each table and figure below present optimal hyper-parameters and learning curves per machine learning technique proposed for each hydraulic component being presented. In addition, the confusion matrix for feature selection for each hydraulic system component, the accuracy determined by the grid search evaluation criteria for the training set, and accuracy, precision, recall (TPR), and TNR confirmed by the testing set are presented. Table 5 shows optimal hyper-parameters obtained by executing feature selection-based machine learning using a grid search for each hydraulic component. The optimal combination was found in the search space of the given hyper-parameters. And Table 6 shows confusion matrix and classification evaluation metrics from each machine learning method configured based on the optimal hyper-parameters proposed in Table 5. Similarly, Table 7 and Table 8, Table 9 and Table 10, and Table 11 and Table 12 show results related to valve condition, internal pump leakage, and hydraulic accumulator, respectively.

**Figure 5:**
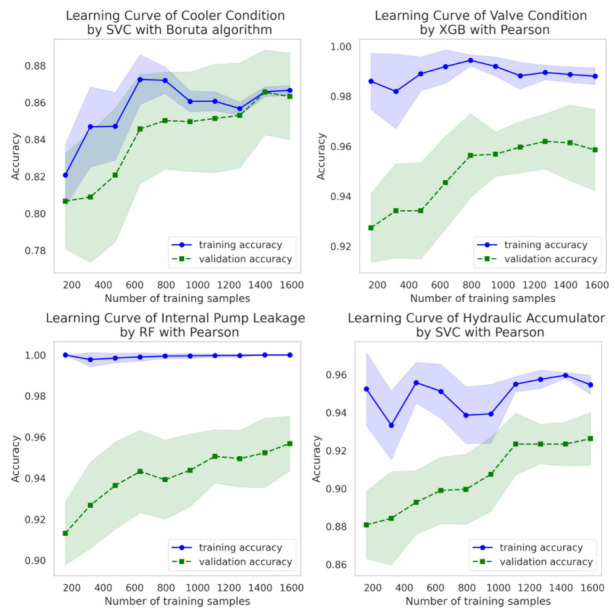
Learning curve of hydraulic component in machine learning.

Figure 5 presents a learning curve to determine whether overfitting or underfitting occurs while learning hyper-parameters for each hydraulic component. Overall, as the number of samples increases, a model with high variance becomes a model with a good bias-variance trade-off. Random forest or XGBoost ML methods tend to have a smaller variance as the number of samples increases. Therefore, if the number of samples can be increased, the variance can be made smaller, making it a model with a good bias-variance trade-off.

Table 5 shows the optimal hyper-parameters obtained by running machine learning based on feature selection using a grid search for cooler condition. All machine learning methodologies were constructed by feature selection through the Boruta algorithm in cooler condition. Table 6 shows the results of the confusion matrix and classification evaluation index for the cooler condition among hydraulic system components. The cooler condition showed the worst classification performance result compared to other components. Furthermore, the classification accuracy was as high as 0.885, indicating the worst classification accuracy among hydraulic components. In particular, the linear discriminant analysis showed the worst performance at 0.655.

The recall performance index also showed the worst classification result in the linear discriminant analysis. The feature selection with the best performance was Spearman’s rank correlation coefficient, which properly classified most of the actual stable states of 0.997, but the TNR was 0.099 in the Boruta algorithm. Additionally, in logistic regression, TPR showed the highest performance for all feature selections, but TNR was zero or 0.006, so it could not detect anomalies. Compared to the recall performance, it showed incomparably poor performance.

In the model with the best recall performance, the SVC using Spearman’s rank correlation coefficient classified the stable state perfectly, but the TNR was 0.093, showing a performance not significantly different from that of the linear discriminant analysis. The models with the best TNR performance were random forest, XGBoost, and LightGBM using the Boruta algorithm. Considering recall and the TNR performance (0.948, 0.762), (0.938, 0.768), (0.935, 0.788), the model that could best judge the cooler condition was selected.

**Table 5:** Hyper-parameter configuration space and optimal parameter under cooler condition.

Hydraulic Component	ML Model (with Feature Selection)	Hyper-Parameter	Type	Search Space	Optimal Parameter
Cooler Condition	LDA (Boruta)	-	-	-	-
	Logistic Reg. (Boruta)	C	Discrete	0.001, 0.01, 0.1, 1, 10, 100	100
		penalty	Categorical	‘L1’, ‘L2’, ‘elasticnet’	L2
	SVC (Boruta)	C	Discrete	0.01, 0.1, 1, 10, 50	50
		kernel	Categorical	‘poly’, ‘rbf’, ‘sigmoid’	rbf
	Decision Tree (Boruta)	max_features	Categorical	‘auto’, ‘sqrt’, ’log2’	auto
		ccp_alpha	Discrete	0.1, 0.01, 0.001	0.001
		max_depth	Discrete	5, 7, 9	5
		criterion	Categorical	‘gini’, ‘entropy’	entropy
	Random Forest (Boruta)	n_estimators	Discrete	10, 100, 1000	100
		max_features	Categorical	‘sqrt’, ‘log2’	sqrt
	XGBoost (Boruta)	gamma	Discrete	0.5, 1, 2	1
		learning_rate	Discrete	0.01, 0.1	0.01
		max_depth	Discrete	3, 4, 5, 6	6
		n_estimators	Discrete	12, 24, 36	12
	LightGBM (Boruta)	num_iterations	Discrete	100, 500, 1000	500
		max_depth	Discrete	15, 20, 25	15
		learning_rate	Discrete	0.01, 0.1, 0.3	0.1
	Multilayer Perceptron (Boruta)	hidden_layer_sizes	Tuple	(50,50,50), (50,100,50), (100,)	(50,100,50)
		activation	Categorical	‘tanh’, ‘relu’	relu
		solver	Categorical	‘sgd’, ‘adam’	adam
		alpha	Discrete	0.0001, 0.05	0.0001
		learning_rate	Categorical	‘constant’, ‘adaptive’	constant

**Table 6:** Classification evaluation metrics for cooler condition.

Cooler Condition			Spearman		Pearson		Boruta	
			Predicted		Predicted		Predicted	
			Stable	Unstable	Stable	Unstable	Stable	Unstable
LDA	True	Stable	289	1	279	11	274	16
		Unstable	150	1	149	2	136	15
	Train Accuracy		0.652		0.655		0.642	
	Test Accuracy		0.658		0.637		0.655	
	Precision		0.658		0.652		0.668	
	Recall (TPR)		0.997		0.962		0.945	
	True Negative Rate		0.007		0.013		0.099	
Logistic Regression	True	Stable	290	0	290	0	290	0
		Unstable	151	0	151	0	144	7
	Train Accuracy		0.567		0.657		0.664	
	Test Accuracy		0.658		0.658		0.673	
	Precision		0.658		0.658		0.668	
	Recall (TPR)		1.000		1.000		1.000	
	True Negative Rate		0.000		0.000		0.046	
SVC	True	Stable	290	0	287	3	253	37
		Unstable	137	14	110	41	38	113
	Train Accuracy		0.684		0.741		0.862	
	Test Accuracy		0.689		0.744		0.830	
	Precision		0.679		0.723		0.869	
	Recall (TPR)		1.000		0.990		0.872	
	True Negative Rate		0.093		0.272		0.748	
Decision Tree	True	Stable	260	30	277	13	271	19
		Unstable	79	72	82	69	34	117
	Train Accuracy		0.722		0.766		0.889	
	Test Accuracy		0.753		0.785		0.880	
	Precision		0.767		0.772		0.889	
	Recall (TPR)		0.897		0.955		0.934	
	True Negative Rate		0.477		0.457		0.775	
Random Forest	True	Stable	263	27	264	26	275	15
		Unstable	75	76	65	86	36	115
	Train Accuracy		0.754		0.773		0.909	
	Test Accuracy		0.769		0.794		0.884	
	Precision		0.778		0.802		0.884	
	Recall (TPR)		0.907		0.910		0.948	
	True Negative Rate		0.503		0.570		0.762	
XGBoost	True	Stable	264	26	274	16	272	18
		Unstable	84	67	72	79	35	116
	Train Accuracy		0.748		0.782		0.899	
	Test Accuracy		0.751		0.800		0.880	
	Precision		0.759		0.792		0.886	
	Recall (TPR)		0.910		0.945		0.938	
	True Negative Rate		0.444		0.523		0.768	
LightGBM	True	Stable	252	38	254	36	272	19
		Unstable	72	79	67	84	32	119
	Train Accuracy		0.762		0.774		0.896	
	Test Accuracy		0.751		0.766		0.885	
	Precision		0.778		0.791		0.895	
	Recall (TPR)		0.869		0.876		0.935	
	True Negative Rate		0.523		0.556		0.788	
Multi-layer Perceptron	True	Stable	290	0	265	25	272	18
		Unstable	137	14	74	77	42	109
	Train Accuracy		0.685		0.76		0.853	
	Test Accuracy		0.689		0.776		0.864	
	Precision		0.679		0.782		0.866	
	Recall (TPR)		1.000		0.914		0.938	
	True Negative Rate		0.093		0.510		0.722	

Table 7 shows the optimal hyper-parameters obtained by running machine learning based on feature selection using a grid search for valve condition. Most of the machine learning methodologies consisted of feature selection through Pearson correlation coefficient in valve condition, and in particular, the LDA model was trained using Spearman’s rank correlation coefficient. Table 8 shows the results of the confusion matrix and classification evaluation index of valve condition among hydraulic system components. Among the models that can determine the state of the valve condition, the linear discriminant analysis had significantly improved performance compared to the result in the cooler condition, but its performance was not as good as that of random forest and XGBoost.

XGBoost showed the best classification accuracy with LDA 0.889, SVM 0.949, random forest 0.966, and XGBoost 0.967. In particular, the feature selection model with Boruta algorithm showed good performance in RF and XGBoost.

In the case of recall performance, LDA 0.86 and SVC 0.94 had the maximum recall performance, and random forest and XGBoost showed performance above 0.95. In particular, the feature selection model with the Boruta algorithm showed a performance of 0.97 or higher.

In the case of TNR performance, the feature selection model with the Boruta algorithm showed overall excellent performance. In particular, 0.98 in LDA and 0.92 in random forest and XGBoost showed excellent performance in LDA.

TPR and TNR of the random forest using Spearman’s rank correlation coefficient and Boruta algorithm for feature selection within the valve condition showed the best performance with (0.972, 0.927) and (0.976, 0.927), respectively. XGBoost using the Boruta algorithm (0.969, 0.921) showed similar performance to the random forest.

**Table 7:** Hyper-parameter configuration space and optimal parameter under valve condition.

Hydraulic Component	ML Model (with Feature Selection)	Hyper-Parameter	Type	Search Space	Optimal Parameter
Valve Condition	LDA (Spearman)	-	-	-	-
	Logistic Reg. (Pearson)	C	Discrete	0.001, 0.01, 0.1, 1, 10, 100	100
		penalty	Categorical	‘L1’, ‘L2’, ‘elasticnet’	L2
	SVC (Pearson)	C	Discrete	0.01, 0.1, 1, 10, 50	50
		kernel	Categorical	‘poly’, ‘rbf’, ‘sigmoid’	rbf
	Decision Tree (Pearson)	max_features	Categorical	‘auto’, ‘sqrt’, ’log2’	auto
		ccp_alpha	Discrete	0.1, 0.01, 0.001	0.001
		max_depth	Discrete	5, 7, 9	7
		criterion	Categorical	‘gini’, ‘entropy’	entropy
	Random Forest (Boruta)	n_estimators	Discrete	10, 100, 1000	100
		max_features	Categorical	‘sqrt’, ‘log2’	sqrt
	XGBoost (Pearson)	gamma	Discrete	0.5, 1, 2	0.5
		learning_rate	Discrete	0.01, 0.1	0.1
		max_depth	Discrete	3, 4, 5, 6	6
		n_estimators	Discrete	12, 24, 36	36
	LightGBM (Boruta)	num_iterations	Discrete	100, 500, 1000	1000
		max_depth	Discrete	15, 20, 25	15
		learning_rate	Discrete	0.01, 0.1, 0.3	0.01
	Multilayer Perceptron (Pearson)	hidden_layer_sizes	Tuple	(50,50,50), (50,100,50), (100,)	(50,100,50)
		activation	Categorical	‘tanh’, ‘relu’	tanh
		solver	Categorical	‘sgd’, ‘adam’	adam
		alpha	Discrete	0.0001, 0.05	0.0001
		learning_rate	Categorical	‘constant’, ‘adaptive’	constant

**Table 8:** Classification evaluation metrics for valve condition.

Valve Condition			Spearman		Pearson		Boruta	
			Predicted		Predicted		Predicted	
			Stable	Unstable	Stable	Unstable	Stable	Unstable
LDA	True	Stable	247	43	250	40	231	59
		Unstable	6	145	11	140	3	148
	Train Accuracy		0.899		0.913		0.858	
	Test Accuracy		0.889		0.884		0.859	
	Precision		0.976		0.958		0.987	
	Recall (TPR)		0.852		0.862		0.797	
	True Negative Rate		0.960		0.927		0.980	
Logistic Regression	True	Stable	260	30	260	30	252	38
		Unstable	10	141	10	141	6	145
	Train Accuracy		0.916		0.926		0.899	
	Test Accuracy		0.909		0.909		0.900	
	Precision		0.963		0.963		0.977	
	Recall (TPR)		0.897		0.897		0.869	
	True Negative Rate		0.934		0.934		0.960	
SVC	True	Stable	267	23	272	18	267	23
		Unstable	13	138	8	143	9	142
	Train Accuracy		0.935		0.949		0.935	
	Test Accuracy		0.918		0.941		0.927	
	Precision		0.954		0.971		0.967	
	Recall (TPR)		0.921		0.938		0.921	
	True Negative Rate		0.914		0.947		0.940	
Decision Tree	True	Stable	274	16	279	11	271	19
		Unstable	18	133	13	138	34	117
	Train Accuracy		0.946		0.955		0.951	
	Test Accuracy		0.923		0.946		0.880	
	Precision		0.938		0.955		0.889	
	Recall (TPR)		0.945		0.962		0.934	
	True Negative Rate		0.881		0.914		0.775	
Random Forest	True	Stable	282	8	284	9	283	7
		Unstable	11	140	12	139	11	140
	Train Accuracy		0.960		0.966		0.966	
	Test Accuracy		0.957		0.959		0.959	
	Precision		0.962		0.959		0.963	
	Recall (TPR)		0.972		0.979		0.976	
	True Negative Rate		0.927		0.921		0.927	
XGBoost	True	Stable	278	12	282	8	281	9
		Unstable	14	137	8	143	12	139
	Train Accuracy		0.963		0.961		0.967	
	Test Accuracy		0.941		0.964		0.952	
	Precision		0.952		0.972		0.959	
	Recall (TPR)		0.959		0.972		0.969	
	True Negative Rate		0.907		0.947		0.921	
LightGBM	True	Stable	280	10	282	8	282	8
		Unstable	11	140	11	140	11	140
	Train Accuracy		0.963		0.967		0.968	
	Test Accuracy		0.952		0.957		0.957	
	Precision		0.962		0.962		0.962	
	Recall (TPR)		0.966		0.972		0.972	
	True Negative Rate		0.927		0.927		0.927	
Multi-layer Perceptron	True	Stable	263	27	279	11	252	38
		Unstable	11	140	13	138	8	143
	Train Accuracy		0.929		0.948		0.931	
	Test Accuracy		0.914		0.946		0.896	
	Precision		0.960		0.955		0.969	
	Recall (TPR)		0.907		0.962		0.869	
	True Negative Rate		0.927		0.914		0.947	

Table 9 shows the optimal hyper-parameters obtained by running machine learning based on feature selection using a grid search for internal pump leakage. Unlike logistic regression using the Boruta algorithm in the case of internal pump leakage, all other machine learning methodologies were learned based on the Pearson correlation coefficient. Table 10 shows the confusion matrix and classification evaluation index of internal pump leakage. In the case of classification accuracy, the random forest with feature selection with Pearson correlation coefficient showed the best performance with 0.961. Regarding LDA feature selection with Spearman’s rank correlation coefficient, 0.832 showed the worst performance.

In the case of recall, the model with the highest classification accuracy also showed the highest recall performance of 0.986. In particular, random forest and XGBoost showed high recall performance, but random forest slightly outperformed them. Moreover, with TNR, as with recall, the random forest showed 0.901 performance overall. Logistic regression showed the highest TNR performance with 0.960, but TPR was 0.869, which was inferior to random forest. Therefore, in the third hydraulic system component, the random forest using the Pearson correlation coefficient for internal pump leakage showed the best performance of (0.986, 0.901) for each TPR and TNR. The XGBoost and logistic regression feature selected with the Boruta algorithm showed the best performance with (0.983, 0.894) and (0.869, 0.960). The performance fell short of that of the random forest.

**Table 9:** Hyper-parameter configuration space and optimal parameter under internal pump leakage.

Hydraulic Component	ML Model (with Feature Selection)	Hyper-Parameter	Type	Search Space	Optimal Parameter
Internal Pump Leakage	LDA (Pearson)	-	-	-	-
	Logistic Reg. (Boruta)	C	Discrete	0.001, 0.01, 0.1, 1, 10, 100	100
		penalty	Categorical	‘L1’, ‘L2’, ‘elasticnet’	L2
	SVC (Pearson)	C	Discrete	0.01, 0.1, 1, 10, 50	50
		kernel	Categorical	‘poly’, ‘rbf’, ‘sigmoid’	rbf
	Decision Tree (Pearson)	max_features	Categorical	‘auto’, ‘sqrt’, ’log2’	auto
		ccp_alpha	Discrete	0.1, 0.01, 0.001	0.001
		max_depth	Discrete	5, 7, 9	9
		criterion	Categorical	‘gini’, ‘entropy’	gini
	Random Forest (Pearson)	n_estimators	Discrete	10, 100, 1000	100
		max_features	Categorical	‘sqrt’, ‘log2’	sqrt
	XGBoost (Pearson)	gamma	Discrete	0.5, 1, 2	2
		learning_rate	Discrete	0.01, 0.1	0.1
		max_depth	Discrete	3, 4, 5, 6	6
		n_estimators	Discrete	12, 24, 36	36
	LightGBM (Pearson)	num_iterations	Discrete	100, 500, 1000	500
		max_depth	Discrete	15, 20, 25	2520
		learning_rate	Discrete	0.01, 0.1, 0.3	0.3
	Multilayer Perceptron (Pearson)	hidden_layer_sizes	Tuple	(50,50,50), (50,100,50), (100,)	(50,100,50)
		activation	Categorical	‘tanh’, ‘relu’	relu
		solver	Categorical	‘sgd’, ‘adam’	adam
		alpha	Discrete	0.0001, 0.05	0.0001
		learning_rate	Categorical	‘constant’, ‘adaptive’	constant

**Table 10:** Classification evaluation metrics for internal pump leakage.

Internal Pump Leakage			Spearman		Pearson		Boruta	
			Predicted		Predicted		Predicted	
			Stable	Unstable	Stable	Unstable	Stable	Unstable
LDA	True	Stable	238	52	250	40	237	53
		Unstable	22	129	15	136	40	111
	Train Accuracy		0.808		0.852		0.785	
	Test Accuracy		0.832		0.875		0.789	
	Precision		0.915		0.943		0.856	
	Recall (TPR)		0.821		0.862		0.817	
	True Negative Rate		0.854		0.901		0.735	
Logistic Regression	True	Stable	241	49	250	40	252	38
		Unstable	31	120	24	127	6	145
	Train Accuracy		0.813		0.855		0.825	
	Test Accuracy		0.819		0.855		0.900	
	Precision		0.886		0.912		0.977	
	Recall (TPR)		0.831		0.862		0.869	
	True Negative Rate		0.795		0.841		0.960	
SVC	True	Stable	246	44	275	15	262	28
		Unstable	11	140	15	136	25	126
	Train Accuracy		0.849		0.922		0.870	
	Test Accuracy		0.875		0.932		0.880	
	Precision		0.957		0.948		0.913	
	Recall (TPR)		0.848		0.948		0.903	
	True Negative Rate		0.927		0.901		0.834	
Decision Tree	True	Stable	283	7	282	8	283	7
		Unstable	25	126	24	127	36	115
	Train Accuracy		0.919		0.931		0.91	
	Test Accuracy		0.927		0.927		0.902	
	Precision		0.919		0.922		0.887	
	Recall (TPR)		0.976		0.972		0.976	
	True Negative Rate		0.834		0.841		0.762	
Random Forest	True	Stable	282	8	282	4	275	15
		Unstable	19	132	13	138	20	131
	Train Accuracy		0.944		0.955		0.914	
	Test Accuracy		0.939		0.961		0.921	
	Precision		0.937		0.957		0.932	
	Recall (TPR)		0.972		0.986		0.948	
	True Negative Rate		0.874		0.901		0.868	
XGBoost	True	Stable	280	10	285	5	274	16
		Unstable	20	131	16	135	23	128
	Train Accuracy		0.938		0.952		0.914	
	Test Accuracy		0.932		0.952		0.912	
	Precision		0.933		0.947		0.923	
	Recall (TPR)		0.966		0.983		0.945	
	True Negative Rate		0.868		0.894		0.848	
LightGBM	True	Stable	281	9	283	7	282	8
		Unstable	12	139	13	138	19	132
	Train Accuracy		0.947		0.964		0.943	
	Test Accuracy		0.952		0.955		0.939	
	Precision		0.959		0.956		0.937	
	Recall (TPR)		0.969		0.976		0.972	
	True Negative Rate		0.921		0.914		0.874	
Multi-layer Perceptron	True	Stable	265	25	265	25	272	18
		Unstable	26	125	13	138	25	126
	Train Accuracy		0.825		0.907		0.9	
	Test Accuracy		0.884		0.914		0.902	
	Precision		0.911		0.953		0.916	
	Recall (TPR)		0.914		0.914		0.938	
	True Negative Rate		0.828		0.914		0.834	

Table 11 shows the optimal hyper-parameters obtained by running machine learning based on feature selection using a grid search for hydraulic accumulator. In the hydraulic accumulator, various feature selections were all used to train the machine learning model. Finally, Table 12 shows the confusion matrix and classification evaluation index of the hydraulic accumulator. For classification accuracy, random forest showed the best performance with LDA 0.878, logistic regression 0.880, SVC 0.930, decision tree 0.909, RF 0.959, XGBoost 0.948, LightGBM 0.980, and MLP 0.923. When all feature selection methods were compared, Spearman’s rank, Pearson correlation coefficient, and Boruta algorithm were 0.980, 0.964, and 0.968, respectively, showing the best performance among classification models by the feature selection method. Particularly, for all of them test accuracy was observed in LightGBM.

Recall showed the best performance of 0.99 or higher in all feature selection methods of random forest. Moreover, for TNR, the SVC obtained by feature selection with the Pearson correlation coefficient was 0.894, showing the best performance. In the random forest, the feature selection model with Boruta algorithm showed slightly lower performance with 0.887. Therefore, random forest and XGBoost using Boruta algorithm for feature selection indicated performances of (0.997, 0.887) and (0.990, 0.868), respectively. In particular, the SVC and LightGBM of the Pearson correlation coefficient, which had the highest TNR performance, was (0.931, 0.894) and (0.986, 0.921), indicating satisfactory performance.

**Table 11:** Hyper-parameter configuration space and optimal parameter under hydraulic accumulator.

Hydraulic Component	ML Model (with Feature Selection)	Hyper-Parameter	Type	Search Space	Optimal Parameter
Hydraulic Accumulator	LDA (Pearson)	-	-	-	-
	Logistic Reg. (Spearman)	C	Discrete	0.001, 0.01, 0.1, 1, 10, 100	100
		penalty	Categorical	‘L1’, ‘L2’, ‘elasticnet’	L2
	SVC (Pearson)	C	Discrete	0.01, 0.1, 1, 10, 50	50
		kernel	Categorical	‘poly’, ‘rbf’, ‘sigmoid’	poly
	Decision Tree (Pearson)	max_features	Categorical	‘auto’, ‘sqrt’, ’log2’	auto
		ccp_alpha	Discrete	0.1, 0.01, 0.001	0.001
		max_depth	Discrete	5, 7, 9	9
		criterion	Categorical	‘gini’, ‘entropy’	gini
	Random Forest (Boruta)	n_estimators	Discrete	10, 100, 1000	1000
		max_features	Categorical	‘sqrt’, ‘log2’	sqrt
	XGBoost (Boruta)	gamma	Discrete	0.5, 1, 2	2
		learning_rate	Discrete	0.01, 0.1	0.1
		max_depth	Discrete	3, 4, 5, 6	6
		n_estimators	Discrete	12, 24, 36	36
	LightGBM(Spearman)	num_iterations	Discrete	100, 500, 1000	500
		max_depth	Discrete	15, 20, 25	20
		learning_rate	Discrete	0.01, 0.1, 0.3	0.3
	Multilayer Perceptron (Pearson)	hidden_layer_sizes	Tuple	(50,50,50), (50,100,50), (100,)	(50,100,50)
		activation	Categorical	‘tanh’, ‘relu’	relu
		solver	Categorical	‘sgd’, ‘adam’	adam
		alpha	Discrete	0.0001, 0.05	0.05
		learning_rate	Categorical	‘constant’, ‘adaptive’	constant

**Table 12:** Classification evaluation metrics for hydraulic accumulator.

Hydraulic Accumulator			Spearman		Pearson		Boruta	
			Predicted		Predicted		Predicted	
			Stable	Unstable	Stable	Unstable	Stable	Unstable
LDA	True	Stable	264	26	263	27	249	41
		Unstable	29	122	27	124	39	112
	Train Accuracy		0.879		0.870		0.816	
	Test Accuracy		0.875		0.878		0.819	
	Precision		0.901		0.907		0.865	
	Recall (TPR)		0.910		0.907		0.859	
	True Negative Rate		0.808		0.821		0.742	
Logistic Regression	True	Stable	266	24	266	24	256	34
		Unstable	29	122	31	120	34	117
	Train Accuracy		0.897		0.88		0.826	
	Test Accuracy		0.880		0.875		0.846	
	Precision		0.902		0.896		0.883	
	Recall (TPR)		0.917		0.917		0.883	
	True Negative Rate		0.808		0.795		0.775	
SVC	True	Stable	280	10	270	20	280	10
		Unstable	21	130	16	135	26	125
	Train Accuracy		0.929		0.930		0.933	
	Test Accuracy		0.930		0.918		0.918	
	Precision		0.930		0.944		0.915	
	Recall (TPR)		0.966		0.931		0.966	
	True Negative Rate		0.861		0.894		0.828	
Decision Tree	True	Stable	278	12	275	15	283	7
		Unstable	32	119	25	126	36	115
	Train Accuracy		0.908		0.909		0.94	
	Test Accuracy		0.900		0.909		0.902	
	Precision		0.897		0.917		0.887	
	Recall (TPR)		0.959		0.948		0.976	
	True Negative Rate		0.788		0.834		0.762	
Random Forest	True	Stable	289	1	287	3	289	1
		Unstable	25	126	19	132	17	134
	Train Accuracy		0.956		0.951		0.956	
	Test Accuracy		0.941		0.950		0.959	
	Precision		0.920		0.938		0.944	
	Recall (TPR)		0.997		0.990		0.997	
	True Negative Rate		0.834		0.874		0.887	
XGBoost	True	Stable	289	1	285	5	287	3
		Unstable	26	125	24	127	20	131
	Train Accuracy		0.952		0.937		0.956	
	Test Accuracy		0.939		0.934		0.948	
	Precision		0.917		0.922		0.935	
	Recall (TPR)		0.997		0.983		0.990	
	True Negative Rate		0.828		0.841		0.868	
LightGBM	True	Stable	290	0	286	4	290	0
		Unstable	9	142	12	139	14	137
	Train Accuracy		0.969		0.968		0.972	
	Test Accuracy		0.980		0.964		0.968	
	Precision		0.970		0.960		0.954	
	Recall (TPR)		1.000		0.986		1.000	
	True Negative Rate		0.940		0.921		0.907	
Multi-layer Perceptron	True	Stable	282	8	279	11	265	25
		Unstable	31	120	23	128	23	128
	Train Accuracy		0.912		0.921		0.922	
	Test Accuracy		0.912		0.923		0.891	
	Precision		0.901		0.924		0.920	
	Recall (TPR)		0.972		0.962		0.914	
	True Negative Rate		0.795		0.848		0.848	

## 5. Discussion

In this study, the model’s performance was judged through classification accuracy by hydraulic component and the TPR and TNR. Regarding classification accuracy, the classification performance of cooler, valve, pump, and accumulator in related studies had performances of 1, 1, 0.80, and 0.65, respectively [18]; however, the results of this study showed that the maximum classification performances of 0.88, 0.96, 0.96, and 0.96 were obtained. It had high classification accuracy in the case of internal pump leakage and hydraulic accumulator. So, in this paper, it had accurate performance, but in the cooler condition, classification accuracy was lower than that of related studies, so it needs to be supplemented in future studies.

In the case of TPR and TNR, the TPR in the related study was 0.97 [15], and the results of this study showed similar performance, with more than 0.97. In particular, this study shows the identification performance of stable and unstable states by additionally calculating a TNR of 0.78 in the cooler condition and a TNR of 0.87 or more in the remaining components.

Furthermore, after feature extraction, classification was performed based on the top 20 features selected by feature selection, but it was not known which features contributed more and played a decisive role in prediction. In future studies, it is expected that explainable artificial intelligence (XAI) will be used to measure the importance of a feature, and it will be used to determine anomalies and problems by combining them with industry domain information based on the importance of features used when anomalies or problems occur in actual industrial sites. It will also be helpful in organizing target variables and metadata of the industrial internet of things (IoT) data collected for the first time.

However, the proposed approach in this paper can be a guide to the occurrence of abnormal conditions for each hydraulic element by using machine learning techniques. Even so, a disadvantage is that the analysis to help the decision-maker according to the engineer’s report is insufficient. Therefore, future research using explainable AI is expected so that engineers can understand the results created by machine learning techniques. Additionally, although the time domain exists, since it is experimental data repeatedly measured according to a certain time, a time series analysis approach cannot be attempted, which is a limitation. Therefore, it is expected that anomaly detection will be the subject of future research by approaching time-series analysis based on repeated measurement experimental data.

## 6. Conclusions

This study deals with the identification of stable and unstable conditions that may occur in hydraulic system components. After classifying data based on a predetermined cluster, features recombined with the shape and distribution density characteristics of observations were extracted. In total 2335 features were used for model learning by selecting variables with high probability due to a high correlation coefficient or high hit with hydraulic system components using a correlation coefficient and Boruta algorithm. Based on the selected features using each feature selection method, the cooler condition, valve condition, internal pump leakage, and stable/unstable condition of the hydraulic accumulator were classified.

The recall rate of all hydraulic system components was 0.94 or higher, and satisfactory results were obtained, but the TNR showed 0.77 in cooler condition. Except for the cooler condition, all components showed a TNR of 0.85 or more, and in particular, the TNR of the valve condition was 0.92, which is very high.

The objective and contributions made through this study are as follows.

- When learning machine learning or deep learning models, it aims to create a new combination of features with feature extraction and to propose better performance models and various classification evaluation scales by reducing the calculation and feature dimensions required for learning the model through feature selection.
- Suggesting TNR to reduce the issue of unexpected system shutdown except for in case of the unstable state of each hydraulic component.
- Justification for the engineer’s judgment by proposing the basis for determining the unstable condition of each hydraulic component.

In addition, in future studies, more specific and interpretive grounds for judgment of engineers and decision makers will be specified using XAI.

TPR and TNR, which were proposed as a result of this paper, can be utilized as a basis for engineers to determine anomalous occurrences and as a factor for decision makers to make situational judgments. Adding this study’s results to the domain used to judge abnormalities and problems in the industrial field can provide a more reliable basis for analysis, and it is expected that the results of XAI in future studies will be added to make a more precise value judgment.

## Figures and Tables

**Table 1:** Condition hydraulic components and stable flag expression in the hydraulic system.

Hydraulic Components	Annotations	Condition Values	Examples	Stable Flag (with Examples)
Cooler Condition	Close to Total Failure	3%	732	0: Conditions were stable (7449). 1: Static conditions might not have been reached yet (756).
	Reduced Efficiency	20%	732	
	Full Efficiency	100%	741	
Valve Condition	Optimal Switching Behavior	100%	1125	
	Small Lag	90%	360	
	Severe Lag	80%	360	
	Close to Total Failure	73%	360	
Internal Pump Leakage	No Leakage	0	1221	
	Weak Leakage	1	492	
	Severe Leakage	2	492	
Hydraulic Accumulator	Optimal Pressure	130 bar	599	
	Slightly Reduced Pressure	115 bar	399	
	Severely Reduced Pressure	100 bar	399	
	Close to Total Failure	90 bar	808	

**Table 2:** Sensor data measured each minute in the hydraulic system.

Category	Sensor	Unit	Sampling Rate (Hz)
Pressure	PS1	Bar	100 Hz
	PS2		
	PS3		
	PS4		
	PS5		
	PS6		
Motor Power	EPS1	W	
Volume Flow	FS1	L/min	10 Hz
	FS2		
Temperature	TS1	°C	1 Hz
	TS2		
	TS3		
	TS4		
Vibration	VS1	mm/s	
Cooling Efficiency	CE	%	
Cooling Power	CP	kW	

**Table 3:** Feature selection through Spearman’s rank correlation coefficient.

Cooler Condition		Valve Condition		Internal Pump Leakage		Hydraulic Accumulator	
Sensor	|r|	Sensor	|r|	Sensor	|r|	Sensor	|r|
CP_min_1	0.942	FS1_maxloc_4	0.946	SE_min_5	0.893	EPS1_var_2	0.788
CP_min_10	0.942	PS2_ma_loc_4	0.944	SE_Q1_5	0.893	EPS1_slope_2	0.774
CP_min_11	0.942	PS3_maxloc_4	0.930	SE_mean_5	0.893	FS1_slope_8	0.759
CP_Q1_10	0.942	PS3_maxloc_1	0.930	SE_median_5	0.892	PS3_Q3_4	0.755
CP_median_10	0.942	PS2_slope_4	0.926	SE_min_13	0.892	FS1_var_10	0.724
CP_mean_10	0.942	PS2_skew_4	0.926	SE_Q1_13	0.892	PS1_var_2	0.697
CP_Q1_1	0.942	PS2_kurt_4	0.926	SE_Q3_1	0.892	PS3_var_4	0.678
CP_Q3_10	0.942	PS1_kurt_4	0.926	SE_mean_13	0.892	EPS1_var_3	0.661
CP_max_11	0.942	PS2_var_4	0.926	SE_median_13	0.891	PS1_var_5	0.656
CP_max_10	0.942	PS1_skew_4	0.926	SE_Q3_5	0.891	FS1_skew_6	0.649
CP_mean_11	0.942	EPS1_skew_4	0.926	SE_Q3_13	0.891	FS1_kurt_6	0.640
CP_Q1_11	0.942	PS2_mean_4	0.906	FS1_Q1_13	0.888	PS1_Q3_2	0.630
CP_min_13	0.942	FS1_kurt_4	0.896	SE_max_13	0.888	PS3_maxloc_2	0.615
CP_median_11	0.942	PS1_Q3_4	0.882	SE_max_5	0.887	EPS1_slope_8	0.607
CP_max_12	0.942	PS2_maxloc_1	0.870	FS1_min_13	0.880	FS1_slope_10	0.605
CP_Q3_11	0.942	PS3_slope_4	0.865	FS1_mean_13	0.876	PS2_var_5	0.602
CP_Q3_12	0.942	FS1_var_4	0.860	FS1_median_13	0.875	FS1_var_8	0.601
CP_min_9	0.942	PS3_Q1_4	0.849	SE_var_2	0.873	EPS1_var_8	0.600
CP_median_12	0.942	FS1_maxloc_1	0.839	SE_mean_2	0.873	PS1_skew_2	0.586
CP_mean_12	0.942	PS1_slope_4	0.828	SE_Q3_2	0.873	EPS1_skew_3	0.580

**Table 4:** Feature selection through Pearson correlation coefficient.

Cooler Condition		Valve Condition		Internal Pump Leakage		Hydraulic Accumulator	
Sensor	|ρ|	Sensor	|ρ|	Sensor	|ρ|	Sensor	|ρ|
CE_Q1_13	0.993	PS2_kurt_4	0.999	SE_min_5	0.951	EPS1_var_2	0.689
CE_min_13	0.993	PS2_skew_4	0.993	SE_Q1_5	0.950	PS3_var_4	0.655
CE_mean_13	0.993	PS2_slope_4	0.991	SE_median_5	0.947	PS3_maxloc_2	0.626
CE_median_13	0.993	PS2_maxloc_4	0.982	SE_mean_5	0.946	PS3_max_3	0.583
CE_Q3_13	0.993	PS3_maxloc_4	0.977	SE_Q3_5	0.940	PS3_Q3_4	0.582
CE_min_12	0.993	PS1_skew_4	0.976	SE_max_5	0.920	EPS1_skew_3	0.568
CE_Q1_12	0.993	PS1_kurt_4	0.976	FS1_Q1_5	0.910	PS1_Q3_2	0.556
CE_mean_12	0.993	EPS1_skew_4	0.975	FS1_min_5	0.896	EPS1_slope_2	0.536
CE_median_12	0.993	PS2_var_4	0.974	FS1_Q3_6	0.895	PS1_median_2	0.517
CE_Q3_12	0.993	FS1_maxloc_4	0.957	FS1_mean_5	0.863	FS1_min_6	0.515
CE_max_12	0.993	PS2_mean_4	0.948	FS1_median_5	0.863	PS3_slope_3	0.513
CE_max_13	0.993	PS3_Q1_4	0.940	FS1_Q3_5	0.799	SE_maxloc_4	0.505
CE_min_11	0.993	PS2_Q1_4	0.929	FS1_max_6	0.763	FS1_slope_2	0.503
CE_Q1_11	0.993	PS3_slope_4	0.916	SE_max_4	0.678	FS1_max_2	0.502
CE_mean_11	0.993	PS1_Q3_4	0.915	FS1_max_5	0.665	FS1_kurt_12	0.499
CE_median_11	0.993	FS1_slope_4	0.903	SE_var_2	0.570	FS1_mean_2	0.493
CE_Q3_11	0.993	FS1_kurt_4	0.899	SE_Q3_4	0.520	FS1_min_8	0.491
CE_max_11	0.993	FS1_var_4	0.869	SE_mean_1	0.465	FS1_var_2	0.482
CE_Q1_10	0.992	FS1_Q1_4	0.863	FS1_mean_6	0.438	PS3_var_3	0.472
CE_median_10	0.992	SE_Q1_4	0.848	SE_Q3_1	0.436	FS1_max_4	0.470

## Data Availability

This data can be found here: [https://archive-beta.ics.uci.edu/ml/datasets/condition+monitoring+of+hydraulic+systems] (accessed on 5 February 2022).
